# Prognostication refinement in *NPM1*‐mutated acute myeloid leukemia stratified by *FLT3*‐ITD status with different induction doses of cytarabine

**DOI:** 10.1002/cam4.5704

**Published:** 2023-02-21

**Authors:** Biao Wang, Xiaoying Hua, Jihong Zhang, Weiying Gu, Haiqian Li

**Affiliations:** ^1^ Department of Hematology The Third Affiliated Hospital of Soochow University (The First People's Hospital of Changzhou) Changzhou China; ^2^ Blood Research Laboratory Shengjing Hospital of China Medical University Shenyang China

**Keywords:** acute myeloid leukemia, cytarabine, *FLT3*‐ITD, next‐generation sequencing, *NPM1*, prognosis

## Abstract

**Objective:**

We aimed to retrospectively discern the heterogeneity of outcomes from clinicopathological characteristics and next‐generation sequencing (NGS) data in adult patients with *NPM1‐*mutated (*NPM1* ^mut^) acute myeloid leukemia (AML) induced with standard‐dose (SD, 100–200 mg/m^2^) and intermediate‐dose (ID, 1000–2000 mg/m^2^) cytarabine arabinose (Ara‐C).

**Methods:**

In the entire cohort and *FLT3*‐ITD subgroups, multivariate Logistic and Cox regression analyses were used to analyze the comprehensive complete remission (cCR) rate after one or two induction cycles, event‐free survival (EFS), and overall survival (OS).

**Results:**

Among a total of 203 *NPM1* ^mut^ patients evaluable for clinical outcome, 144 (70.9%) received a first SD‐Ara‐C induction and 59 (29.1%) received ID‐Ara‐C induction. Early death was recorded in seven (3.4%) after one or two cycles of induction. Focusing analysis on the *NPM1* ^mut^/*FLT3*‐ITD^(−)^ subgroup, independent factors showing inferior outcome were presence of *TET2* mutation [cCR rate, OR = 12.82 (95%CI 1.93–85.28), *p* = 0.008; EFS, HR = 2.92 (95%CI 1.46–5.86), *p* = 0.003], increasing age [EFS, HR = 1.49 (95%CI 1.10–2.02), *p* = 0.012 by every 10‐years elevation], white blood cell count ≥60 × 10^9^/L [EFS, HR = 3.30 (95%CI 1.63–6.70), *p* = 0.001], and ≥4 mutated genes at initial diagnosis [OS, HR = 5.54 (95%CI 1.77–17.33), *p* = 0.003]. In contrast, when focusing on the *NPM1* ^mut^/*FLT3*‐ITD^(+)^ subgroup, factors showing superior outcome were ID‐Ara‐C induction [cCR rate, OR = 0.20 (95%CI 0.05–0.81), *p* = 0.025; EFS, HR = 0.27 (95%CI 0.13–0.60), *p* = 0.001] and allo‐transplantation [OS, HR = 0.45 (95%CI 0.21–0.94), *p* = 0.033]. Factors showing inferior outcome included CD34^(+)^ [cCR rate, OR = 6.22 (95%CI 1.86–20.77), *p* = 0.003; EFS, HR = 2.01 (95%CI 1.12–3.61), *p* = 0.020] and ≥5 mutated genes [OS, HR = 2.85 (95%CI 1.33–6.10), *p* = 0.007].

**Conclusion:**

We conclude that *TET2* ^(+)^, age, and white blood cell count convey an outcome risk modulation for AML with *NPM1* ^mut^/*FLT3*‐ITD^(−)^, as does CD34 and ID‐Ara‐C induction for *NPM1* ^mut^/*FLT3*‐ITD^(+)^. The findings permit re‐stratification of *NPM1* ^mut^ AML into distinct prognostic subsets to guide risk‐adapted individualized treatment.

## Graphical abstract

NPM1mut/FLT3‐ITD(−)/TET2(+) is identified as an unfavorable prognostic subset in NPM1mut/FLT3‐ITD(−) patients. Induction containing intermediate‐dose cytosine arabinoside improves outcome in NPM1mut/FLT3‐ITD(+) patients. Clinicopathologic parameters, including age and white blood cell count, convey an outcome risk modulation for NPM1mut/FLT3‐ITD(−), as does CD34 for NPM1mut/FLT3‐ITD(+).

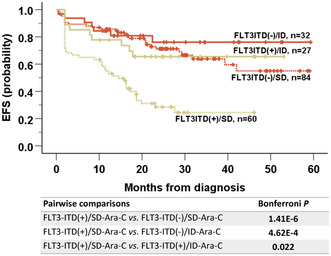

## 1 INTRODUCTION

The human *nucleophosmin 1* (*NPM1*) gene is multifunctional, including chromatin remodeling, ribosome biogenesis, genomic stability, regulation of tumor suppressors, and transcription factors.^1^, ^2^, ^3^ Given its unique biological and clinical relevance, the 2016 revision of World Health Organization (WHO) classification of myeloid neoplasms and acute leukemia recognized *NPM1* ^mut^ acute myeloid leukemia (AML) as a distinct entity.^4^ *NPM1* mutation (*NPM1* ^mut^) is present in approximately one‐third de novo AML, and up to ~60% in normal karyotype (NK) AML.^5^, ^6^ Approximately 40% of *NPM1* ^mut^ cases coexist with the fms‐like tyrosine kinase 3 (*FLT3*)‐internal tandem duplication (ITD). In the latest NCCN guideline^7^ and 2017‐European LeukemiaNet recommendation,^8^ *NPM1* ^mut^ AML is divided into favorable‐ and intermediate‐risk groups according to a presence or absence of concomitant *FLT3*‐ITD and allele ratio (AR) levels. *NPM1* ^mut^ AML with negative *FLT3‐*ITD and *FLT3‐*ITD low AR (*FLT3‐*ITD^low^) genotype is in the favorable‐risk category. Yet, a considerable proportion of these patients experience relapse and resistance, ultimately shortening their survival. Even the outcome of *NPM1* ^mut^/*FLT3*‐ITD^(−)^ patients still has considerable heterogeneity.^9^, ^10^

Standard‐dose (SD) cytarabine arabinoside (Ara‐C) combined with anthracyclines (i.e., “7 + 3” scheme) is the most commonly used AML induction protocol. The literature of high‐dose (HD) Ara‐C used for AML chemotherapy was first published in 1985. Several randomized trials were performed; the majority failed to draw a conclusion of significant efficacy improvement, as reported during 1991–2011.^11^ However, prolonged follow‐up time subsequently identified subsets of patients benefiting from HD‐Ara‐C, like AML with t(8;21)^12^ and Ras‐pathway mutations.^13^ Furthermore, most of the earlier studies did not comprehensively integrate the genomics‐based prognostication scheme. Thus, the conclusions could not reflect the concept of risk‐adapted therapy using HD‐Ara‐C. Development of massively parallel sequencing methodology has increasingly emphasized the risk‐stratification oriented mode of treatment.

It has reported that the cellular uptake of Ara‐C was insufficient in *FLT3*‐ITD^(+)^ K562 cells, with no significant changes of intracellular deoxycytidine kinase (DCK) and cytidine deaminase (CDA) responsible for metabolism of Ara‐C, and transcription level of multidrug resistance protein 1 (MDR1) related to drug efflux.^14^ Another study described that *FLT3*‐ITD decreased the expression of equilibrative nucleoside transporter 1 (ENT1) by upregulating hypoxia inducible factor‐1 alpha subunit (HIF‐1α), compromising Ara‐C uptake by leukemic cells. In addition, *FLT3*‐ITD also drives a specific Ara‐C resistance mechanism via the downstream molecule RUNX3.^15^ In view of the linkage between *FLT3*‐ITD^(+)^ with distinctive Ara‐C insensitivity, we wonder whether increasing Ara‐C in the induction period can improve outcome of *NPM1* ^mut^/*FLT3*‐ITD^(+)^ populations. According to a review regarding dose‐efficacy relationship of Ara‐C, a level of ID‐Ara‐C in each treatment cycle in AML is sufficient, while HD‐Ara‐C seems to be of little significance.^11^

In this study, we retrospectively analyzed the clinical characteristics of *NPM1* ^mut^ patients and data of next‐generation sequencing (NGS) mutation spectrum concerning 112 genes related to blood disease, synthetically including SD‐Ara‐C 100–200 mg/m^2^ or ID‐Ara‐C 1000–2000 mg/m^2^ induction as covariates. We aimed to refine patient subsets carrying potentially poor remission and prognostic characters from this relatively favorable AML subtype, to guide further risk‐adapted treatment.

## 2 PATIENTS AND METHODS

### 2.1 Subject population and workup

We performed a retrospective review of newly diagnosed de novo AML patients in our institute, Shengjing Hospital of China Medical University, from October 2014 to September 2020. Diagnosis of AML fulfilled the WHO criteria,^16^ in which the clinicopathological workup included morphology, immunophenotyping, chromosome karyotyping, and fluorescence in situ hybridization, molecular biology, and gene mutation analysis (see below). According to literature,^17^ approximately 50% of *NPM1* ^mut^ patients display two populations of blasts, i.e., leukemic myeloid blasts and leukemic immature monocytes, in flow cytometry (FCM) two‐dimensional scatterplot at the initial diagnosis especially in the French‐American‐British (FAB) M4/M5 subtype. When disease relapse occurs, leukemic immature monocytes often disappear, implicating leukemic myeloid blasts as the origin of leukemic recurrence. In baseline immunophenotyping, we only characterized the antigen expression profiles of leukemic myeloid blasts. The study was conducted according to the Declaration of Helsinki and was approved by the institutional review boards of all participating hospitals. Written informed consent was obtained from all patients, or their parents or legal guardians for patients aged under 18 years, for receiving therapies and using their records.

### 2.2 NGS

Massively parallel sequencing was performed on MiSeq/HiSeq (Illumina) or Ion torrent PGM™ (Life Technologies) platforms. A custom‐designed panel of oligonucleotide probe was made to capture the exons of 112 potentially mutated genes involved in hematological diseases as previously reported.^18^ Sequencing reads in FASTQ format were aligned to the human reference genome (GRCh38) using Burrows–Wheeler Aligner (BWA, v0.6) and the SAMtools algorithm. Variant calling for somatic alterations, including single nucleotide variants and short fragment indels in protein coding sequence (CDS), were analyzed by using multiple pipelines (Ion Reporter™ and Variant Reporter) and annotated referencing to the dbSNP (Single Nucleotide Polymorphism database), 1000 Genomes, PolyPhen‐2, and COSMIC (Catalogue of Somatic Mutations in Cancer) databases. FLT3‐ITD were also identified and/or confirmed by PCR and capillary electrophoresis, as described previously.^19^

### 2.3 Treatment and response evaluation

Patients received either one of the three regimens as initial induction treatment. The SD‐Ara‐C containing regimen consists of continuous intravenous Ara‐C 100–200 mg/m^2^/day on days 1–7, plus bolus intravenous daunorubicin 45–60 mg/m^2^/day or idarubicin 10–12 mg/m^2^/day on days 1–3. The ID‐Ara‐C containing regimen consists of over 2 hours intravenous Ara‐C 1000 mg/m^2^/day every 12 h on days 5–7, with other agents and schedules the same as those in the SD‐Ara‐C containing regimen. The third induction regimen involved low‐dose moderate schemes, represented by CAG (cytarabine ± aclarubicin + granulocyte‐colony stimulating factor) or CAG‐like regimen ± hypomethylating agents (azacitidine or decitabine). Palliative care was offered to unfit patients of advanced age and frailty. Bone marrow response was assessed between days 21 and 28 after induction upon recovery of peripheral blood (PB) counts. When no PB recovery was noticed or leukemic blasts persisted or reappeared in the PB, the response evaluation was postponed to no later than day 35 after induction.

Patients who achieved comprehensive complete remission (cCR) after one or two cycles of induction received the following consolidation therapies: (i) identical regimen as that initially used to achieve CR, generally as the first post‐remission treatment; (ii) single agent Ara‐C at 3 g/m^2^ every 12 h delivered by an infusion over 3 hours on days 1–3, or on days 1, 3, and 5; (iii) Ara‐C 1–2 g/m^2^ every 12 h by an infusion over 3 hours on days 1–4, coupled with 3 days of daunorubicin, idarubicin, mitoxantrone, or omacetaxine mepesuccinate; or (iv) FLAG regimen comprising fludarabine 25–30 mg/m^2^ on days 2–6, Ara‐C 2 g/m^2^ delivered for over 4 h starting 4 h after fludarabine infusion on days 2–6, and granulocyte‐colony stimulating factor subcutaneously daily on days 1–7. Three to four courses of consolidation as determined by the attending physicians were scheduled if patients maintained CR status. For those with *NPM1* ^mut^/*FLT3*‐ITD^(+)^ or who experienced a second CR, allogenic stem cell transplantation (Allo‐SCT) was implemented depending on the wishes of patients and availability of HLA‐matched donors. If available, the FLT3 inhibitors midostaurin or sorafenib were prescribed for *NPM1* ^mut^/*FLT3*‐ITD^(+)^ patients.

### 2.4 Safety and toxicities

Nadir of absolute neutrophil count (ANC) was defined as the lowest ANC experienced after induction treatment. Duration of neutrophil and platelet recovery were defined as time from start of induction until the first day of ANC >1.0 × 10^9^/L and platelet count >30 × 10^9^/L without subsequent platelet transfusion, respectively. With the exception of CR with incomplete blood count recovery (CRi), only patients achieving CR were included for the analysis of recovery.

### 2.5 Outcome endpoint and definitions

The primary endpoints were cCR rate after one or two induction cycles, event‐free survival (EFS), and overall survival (OS). In this study, CR and CRi were collectively termed CR_(i)_. Definition of outcome endpoints was according to the 2017‐ELN guideline.^8^

### 2.6 Treatment bias test

Prior to outcome analysis, treatment bias test was performed. At each endpoint, SD‐ and ID‐Ara‐C containing induction groups were compared for distribution of baseline variables, as listed in Table 1, with induction and/or induction‐related biased variables as covariates when applicable.

**TABLE 1:** Characteristics of clinicopathological and genetic alterations of *NPM1* ^mut^ AML at initial diagnosis stratified by *FLT3*‐ITD.

Variables	Entire cohort	*NPM1* ^mut^/*FLT3*‐ITD^(−)^	*NPM1* ^mut^/*FLT3*‐ITD^(+)^	*p*
No. of patients	238	143	95	NA
Median age (range), y	49 (15–81)	50 (16–81)	47 (15–65)	0.142
Sex (M:F), *N*	105:133	58:85	47:48	0.175
Median WBC count (range), ×10^9^/L	26.7 (0.7–321.0)	14.0 (0.7–321.0)	42.0 (1.3–183.0)	< ***0.001***
Median Hb level (range), g/L	83 (31–154)	79 (31–137)	87 (49–154)	***0.010***
Median PLT count (range), ×10^9^/L	55.5 (2–504)	59 (2–441)	50 (9–504)	0.102
Median *WT1* ratio (range), %	96.91 (0–704.31)	71.43 (0–425.60)	121.79 (1.16–704.31)	***0.002***
FAB classification, *n* (%)				
M1	6 (2.5)	4 (2.8)	2 (2.1)	1.000C
M2	42 (17.6)	34 (23.8)	8 (8.4)	***0.002***
M4	37 (15.5)	20 (14.0)	17 (17.9)	0.415
M5	142 (59.7)	78 (54.5)	64 (67.4)	***0.048***
M6	2 (0.8)	2 (1.4)	0 (0.0)	0.518F
Unclassifiable	9 (3.8)	5 (3.5)	4 (4.2)	1.000C
Immunophenotype, *n*/*N* (%)^a^				
CD34	74/235 (31.5)	29/141 (20.6)	45/94 (47.9)	< ***0.001***
TdT	16/231 (6.9)	7/139 (5.0)	9/92 (9.8)	0.164
HLA‐DR	157/233 (67.4)	94/139 (67.6)	63/94 (67.0)	0.923
CD117	211/235 (89.8)	127/141 (90.1)	84/94 (89.4)	0.860
CD13	207/233 (88.8)	128/139 (92.1)	79/94 (83.5)	0.056
CD33	233/233 (100.0)	139/139 (100.0)	94/94 (100.0)	NA
CD123	230/232 (99.1)	138/140 (98.6)	92/92 (100.0)	0.519F
CD38	209/233 (89.7)	124/140 (88.6)	85/93 (91.4)	0.487
MPO	170/227 (74.9)	107/136 (78.7)	63/91 (69.2)	0.108
CD11b	39/159 (24.5)	18/88 (20.5)	21/71 (29.6)	0.184
CD15	39/162 (24.1)	28/96 (29.2)	11/66 (16.7)	0.067
CD14	9/112 (8.0)	6/63 (9.5)	3/49 (6.1)	0.759C
CD64	69/206 (33.5)	48/125 (38.4)	21/84 (25.9)	0.064
CD56	44/172 (25.6)	24/108 (22.2)	20/64 (31.3)	0.190
CD9	135/172 (78.5)	77/97 (79.4)	58/75 (77.3)	0.746
CD19	8/288 (3.5)	3/137 (2.2)	5/91 (5.5)	0.337C
CD79a	1/227 (0.4)	0/137 (0)	1/90 (1.1)	0.396F
CD7	94/218 (43.1)	38/127 (29.9)	56/91 (61.5)	< ***0.001***
Cytogenetics, *n*/*N* (%)				
NK	208/234 (88.9)	121/140 (86.4)	87/94 (83.6)	0.144
IRAK	17/234 (7.3)	10/140 (7.1)	7/94 (7.4)	0.930
NIRAK	9/234 (3.8)	9/140 (6.4)	0/94 (0)	***0.031C***
Median *N* (range) of mutated genes	4.5 (2–14)	4 (2–14)	5 (2–10)	0.378
*NPM1* short indels, *n* (%)	230 (96.6)	135 (94.4)	95 (100.0)	***0.048***
*NPM1* point substitution, *n* (%)	10 (4.2)	10 (7.0)	0 (0)	***0.021***
Co‐mutations, *n* (%)				
Signaling pathways				
*NRAS*	40 (16.8)	35 (24.5)	5 (5.3)	< ***0.001***
*KRAS*	16 (6.7)	13 (9.1)	3 (3.2)	0.073
*PTPN11*	30 (12.6)	24 (16.8)	6 (6.3)	***0.017***
*NOTCH2*	14 (5.9)	8 (5.6)	6 (6.3)	0.817
*RELN*	16 (6.7)	9 (6.3)	7 (7.4)	0.746
Epigenetic regulators				
*DNMT3A*	104 (43.7)	52 (36.4)	52 (54.7)	***0.005***
*DNMT3A*‐R882	77 (32.4)	40 (28)	37 (38.9)	0.076
*TET2*	37 (15.5)	21 (14.7)	16 (16.8)	0.653
*IDH1*	30 (12.6)	25 (17.5)	5 (5.3)	***0.005***
*IDH2*	12 (5.0)	7 (4.9)	5 (5.3)	1.000C
*KMT2D*	31 (13.0)	20 (14.0)	11 (11.6)	0.589
*CREBBP*	16 (6.7)	7 (4.9)	9 (9.5)	0.167
Tumor suppressors				
*FAT1*	57 (23.9)	36 (25.2)	21 (22.1)	0.587
*WT1*	15 (6.3)	10 (7.0)	5 (5.3)	0.591
*DIS3*	13 (5.5)	9 (6.3)	4 (4.2)	0.489
Transcription factors				
*CEBPA*	21 (8.8)	14 (9.8)	7 (7.4)	0.519
*CEBPA*dm	4 (1.7)	3 (2.1)	1 (1.1)	0.921C

*Note*: Parameters showing statistical significance are highlighted in bold and italic.

Abbreviations: C, continuity correction; F, female; F, Fisher's exact test; Hb, hemoglobin; IRAK, intermediate‐risk abnormal karyotype; M, male; NA, not applicable; NIRAK, non‐intermediate risk abnormal karyotype; NK, normal karyotype; *p*#, *p*‐values were obtained from *χ* ^2^ test after cross tabulation for categorical variables and the Mann–Whitney *U* test for continuous variables of non‐normal distribution; PLT, platelet; WBC, white blood cell.

^a^ Percentage according to available data.

### 2.7 Statistical analyses and plotting

Descriptive statistics are presented as medians and ranges for non‐normal continuous variables, and as numbers and percentages for categorical variables. The age and white blood cell (WBC) continuous variables were transformed into a dichotomous variable with cutoff value determined referring to the Youden indices of receiver operating characteristics curve using Medcalc software, as well into an ordinal polychotomous variable by an every increment of 10.0 × 10^9^/L for WBC and 10 years for age, respectively. Chi‐square and Mann–Whitney *U* tests were used to calculate statistical significance for categorical and continuous variables, respectively. Survival estimates were calculated with the Kaplan–Meier method and compared with the log‐rank test. In the entire *NPM1* ^mut^ cohort and layers by *FLT3*‐ITD, variables with a *p* < 0.15 from univariate analyses were further included into the multivariate Logistic and Cox model using a stepwise forward selection procedure to determine independent associations with response rate and survival. When covariates encompassed age and/or WBC, their continuous, dichotomous variables along with ordinal polychotomous variables were together examined in a multivariate model. If applicable, the above‐tested induction‐related biased confounders (Tables S1 and S2) were adjusted in a multivariate model. *p* < 0.05 was considered significant. Statistical analyses were performed by IBM SPSS for Windows, version 26.0.

## 3 RESULTS

### 3.1 Selection of patients for outcome analysis

**FIGURE 1:**
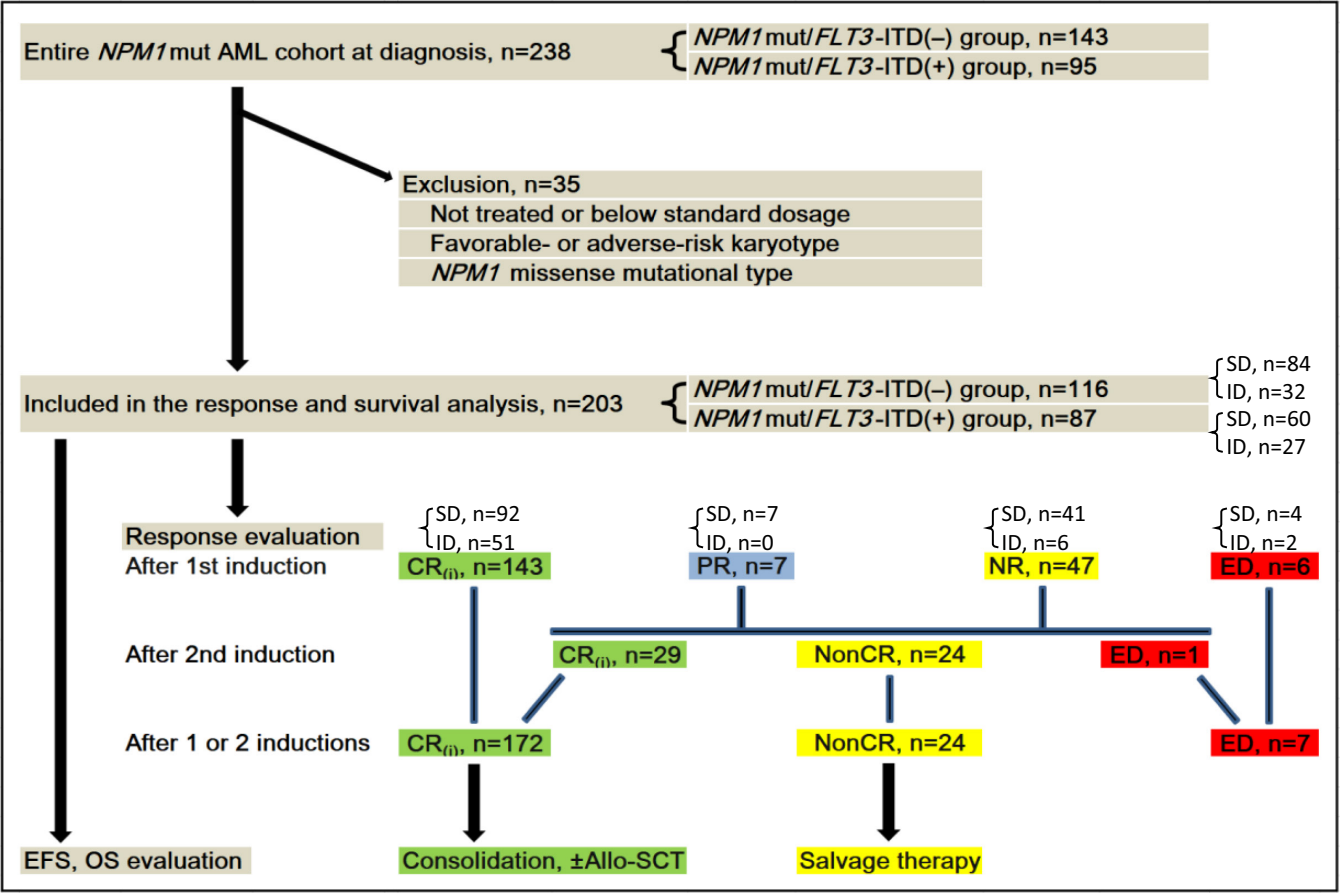
Flowchart of inclusion and exclusion criteria for outcome analysis in *NPM1* ^mut^ AML. CR, complete remission; CRi, CR with incomplete blood count recovery; ED, early death; EFS, event‐free survival; NonCR, no CR, including PR + NR; NR, not remission; OS, overall survival; PR, partial remission.

We selected a total of 238 *NPM1* ^mut^ AML patients (105 men and 133 women; median age, 49 years; range, 15–81 years). Among them, 203 *NPM1* ^mut^ patients who met the following three criteria were included in the remission and prognosis analysis. First, low‐dose and no treatment patients were excluded. Only patients who received SD‐ or ID‐Ara‐C containing induction regimen were included. As of the time of writing, patients attaining CR who were induced with one or two cycles had undergone at least one cycle of ID‐ or HD‐Ara‐C consolidation. Second, favorable‐ and adverse‐risk karyotypes were excluded. Only NK and intermediate‐risk abnormal karyotypes were included. Four patients lack of metaphase were screen by fluorescence in situ hybridization and 43 kinds of fusion transcripts, which ruled out recurrent chromosomal translocations. They were mostly likely NK or intermediate‐risk abnormal karyotypes because of the very low probability of complex or monosomy karyotypes. The four patients also participated in the analysis. Third, *NPM1* ^mut^ missense mutations were excluded. Only those with the insertions/deletions (indels) type were included. Additionally, among 10 *NPM1* ^mut^ missense cases detected in our study, outcome analysis included two cases with *NPM1* ^mut^ missense who each occurred concomitant with a type‐A mutation. The remaining eight cases with *NPM1* ^mut^ missense were excluded. The flowchart of the selected and excluded cases for the outcome analysis is presented in Figure 1.

### 3.2 Baseline characteristics of *NPM1* ^mut^ patients according to *FLT3*‐ITD

As shown in Table 1, there were differences regarding baseline clinicopathological characteristics and NGS genetic profiles for newly diagnosed *NPM1* ^mut^ patients according to *FLT3*‐ITD status. Notably, WBC, CD34, CD7, and coexisting *NRAS* mutations were among the most significant (*p* < 0.001). In particular, the non‐intermediate (high plus low) risk abnormal karyotype was only found in the *NPM1* ^mut^/*FLT3*‐ITD^(−)^ group (9/140, 6.4%) and was absent in the *NPM1* ^mut^/*FLT3*‐ITD^(+)^ group (0%; *p* = 0.031). *NPM1* ^mut^ missense mutations were only found in the *NPM1* ^mut^/*FLT3*‐ITD^(−)^ group (10/143, 7.0%); they were absent in the *NPM1* ^mut^/*FLT3*‐ITD^(+)^ group (0%; *p* = 0.031). Moreover, almost all *NPM1* missense mutations (9/10, 90.0%) were accompanied with an AML subtype‐defining cytogenetic or molecular abnormality, all of which were in the low‐risk (seven cases) or high‐risk category (two cases) (data not shown). Two cases with an *NPM1* ^mut^ missense each occurred concomitant with type‐A mutation.

### 3.3 *TET2* in *NPM1* ^mut^ / *FLT3*‐ITD ^(−)^, and CD34 and ID‐Ara‐C in *NPM1* ^mut^ / *FLT3*‐ITD ^(+)^ influence cCR rate

A total of 203 *NPM1* ^mut^ patients met the requirements of outcome evaluation, as shown in the flowchart (Figure 1). Among the entire cohort, 144 (70.9%) were treated with SD‐Ara‐C and 59 (29.1%) with ID‐Ara‐C. Excluding six cases (3.0%) of early death (ED), the initial overall remission rate after a first induction cycle could be evaluated in 197 cases. Of these, 143 (72.6%) reached CR_(i)_. Among these responders, the incidence of septicemia were analogous in the ID‐ and SD‐Ara‐C induction groups (16/92 vs. 9/51; *p* = 0.969). When confining cases obtaining a CR (excluding CR_i_), there were also no significant differences in the nadir of ANC and platelet, as well as duration of neutrophil and platelet recovery between the two induction groups (data not shown).

After an initial chemotherapy, 54 cases not attaining CR_(i)_ (seven cases with PR and 47 with NR) were administered a second cycle. Out of 53 cases evaluable for response after excluding one case of ED, 29 (54.7%) acquired re‐induction CR_(i)_. As expected, the re‐induction CR_(i)_ rate in the *NPM1* ^mut^/*FLT3*‐ITD^(+)^ group was significantly lower than in the *NPM1* ^mut^/*FLT3*‐ITD^(−)^ group (12/31, 38.7% vs. 17/22, 77.3%; *p* = 0.005). Among the four *NPM1* ^mut^/*FLT3*‐ITD^(+)^ patients who did not attain CR after the first induction cycle of ID‐Ara‐C, re‐induction CR was seen in only one patient, who had obtained a preceding PR.

Thus, in the entire *NPM1* ^mut^ cohort, ED was recorded in 7 of 203 (3.4%) patients after one or two cycles of induction. Among the seven deaths, three were in the *NPM1* ^mut^/*FLT3*‐ITD^(−)^ and four in the *NPM1* ^mut^/*FLT3*‐ITD^(+)^ group, with no significant difference (3/116 vs. 4/87; *p* = 0.698). In the SD‐Ara‐C induction group, the death rate of 3.5% (5/144) was similar to that of 3.4% (2/59) in the ID‐Ara‐C induction group (*p* = 1.000; Figure 1). The remaining 196 patients were evaluated for cCR rate, which was achieved in 172 (87.8%) cases. Of these patients, those in the *NPM1* ^mut^/*FLT3*‐ITD^(−)^ group outmatched the *NPM1* ^mut^/*FLT3*‐ITD^(+)^ group (105/110, 95.5% vs. 67/86, 77.9%; *p* < 0.001).

Logistic analysis of the entire cohort revealed independent factors impacting cCR rate, which included CD34 [hazard ratio (HR) = 4.80 (1.86–12.38), *p* = 0.001] and *FLT3* [HR = 4.54 (1.26–16.40), *p* = 0.021]. Following *FLT3*‐ITD stratification, only *TET2* ^(+)^ was independently associated with a lower cCR rate [HR = 12.82 (1.93–85.28), *p* = 0.008] in the *NPM1* ^mut^/*FLT3*‐ITD^(−)^ group. Among the *NPM1* ^mut^/*FLT3*‐ITD^(+)^ group, CD34^(+)^ was independently associated with a lower cCR rate [HR = 6.22 (1.86–20.77), *p* = 0.003] and ID‐Ara‐C induction with a higher cCR rate [HR = 0.20 (0.05–0.81), *p* = 0.025] (Table 2).

**TABLE 2:** Univariate *χ* ^2^ and multivariate Logistic analysis of cCR rate in *NPM1* ^mut^ patients stratified by *FLT3*‐ITD.

Group	Factors	Versus	*χ* ^2^		Logistic	
			cCR rate (%)	*p*	HR (95%CI)	*p*
Entire cohort^a^	CD34	(−) vs. (+)	129/137 (94.2) vs. 40/56 (71.4)	<0.001	***4.80* (*1.86–12.38*)**	***0.001***
	*FLT3*	(−) vs. (+)	84/87 (96.6) vs. 88/109 (80.7)	0.001	***4.54* (*1.26–16.40*)**	***0.021***
	*FLT3*‐ITD	(−) vs. (+)	105/110 (95.5) vs. 67/86 (77.9)	<0.001	NS	NS
	*DNMT3A*	(−) vs. (+)	98/106 (92.5) vs. 74/90 (82.2)	0.029	NS	NS
*NPM1* ^mut^/*FLT3*‐ITD^(−)^	*TET2*	(−) vs. (+)	94/96 (97.9) vs. 11/14 (78.6)	0.014	***12.82* (*1.93–85.28*)**	***0.008***
*NPM1* ^mut^/*FLT3*‐ITD^(+)^ ^a^	CD34	(−) vs. (+)	40/45 (88.9) vs. 26/40 (65.0)	0.008	***6.22* (*1.86–20.77*)**	***0.003***
	Induction	SD vs. ID	43/59 (72.9) vs. 24/27 (88.9)	0.097	***0.20* (*0.05–0.81*)**	***0.025***

*Note*: Parameters showing statistical significance are highlighted in bold and italic.

Abbreviations: CI, confidence interval; HR, hazard ratio; ID, intermediate‐dose; NS, not significant; SD, standard‐dose.

^a^ Adjusted for induction‐associated biased factors.

### 3.4 *TET2* , age, and WBC in *NPM1* ^mut^ / *FLT3*‐ITD ^(−)^, and CD34 and ID‐Ara‐C in *NPM1* ^mut^ / *FLT3*‐ITD ^(+)^ influence EFS

Overall, independent predictors on EFS included WBC [HR = 2.01 (1.28–3.16), *p* = 0.002], *FLT3*‐ITD [HR = 2.53 (1.61–3.98), *p* < 0.001], and induction [HR = 0.43 (0.25–0.76), *p* = 0.003]. Following *FLT3*‐ITD stratification, *TET2* ^(+)^ [HR = 2.92 (1.46–5.86), *p* = 0.003], age [HR = 1.49 (1.10–2.02), *p* = 0.012 by an every 10‐years increment], and WBC ≥60 × 10^9^/L [HR = 3.30 (1.63–6.70), *p* = 0.001] independently predicted inferior EFS in *NPM1* ^mut^/*FLT3*‐ITD^(−)^ group. In the *NPM1* ^mut^/*FLT3*‐ITD^(+)^ group, CD34^(+)^ [HR = 2.01 (1.12–3.61), *p* = 0.020] independently predicted inferior EFS, and ID‐Ara‐C [HR = 0.27 (0.13–0.60), *p* = 0.001] independently predicted superior EFS (Table 3).

**TABLE 3:** Univariate Kaplan–Meier and multivariate Cox analysis of EFS in *NPM1* ^mut^ patients stratified by *FLT3*‐ITD.

Group	Factors	Versus	Kaplan–Meier	Cox	
			*p*	HR (95%CI)	*p*
Entire cohort^a^	Sex	F vs. M	0.055	NS	NS
	WBC	<60 vs. ≥60	<0.001	***2.01* (*1.28–3.16*)**	***0.002***
	CD34	(−) vs. (+)	0.021	NS	NS
	*FLT3*	(−) vs. (+)	<0.001	NS	NS
	*FLT3*‐ITD	(−) vs. (+)	<0.001	***2.53* (*1.61–3.98*)**	< ***0.001***
	*PTPN11*	(−) vs. (+)	0.068	NS	NS
	*TET2*	(−) vs. (+)	0.033	NS	NS
	Induction	SD vs. ID	0.015	***0.43* (*0.25–0.76*)**	***0.003***
*NPM1* ^mut^/*FLT3*‐ITD^(−)^ ^b^	Age	<50 vs. ≥50	0.066	***1.49* (*1.10–2.02*)**	***0.012*** ^c^
	WBC	<60 vs. ≥60	0.001	***3.30* (*1.63–6.70*)**	***0.001***
	No. of mutated genes	<4 vs. ≥4	0.121	NS	NS
	*TET2*	(−) vs. (+)	<0.001	***2.92* (*1.46–5.86*)**	***0.003***
*NPM1* ^mut^/*FLT3*‐ITD^(+)^ ^d^	CD34	(−) vs. (+)	0.132	***2.01* (*1.12–3.61*)**	***0.020***
	TdT	(−) vs. (+)	0.084	NS	NS
	Induction	SD vs. ID	0.004	***0.27* (*0.13–0.60*)**	***0.001***

*Note*: Parameters showing statistical significance are highlighted in bold and italic.

Abbreviations: CI, confidence interval; F, female; HR, hazard ratio; ID, intermediate‐dose; M, male; NS, not significant; SD, standard‐dose; WBC, white blood cell.

^a^ Adjusted for age and *KTM2D*.

^b^ Adjusted for induction regimen.

^c^ By an every 10‐years increment.

^d^ Adjusted for induction‐associated biased factors.

### 3.5 *NPM1* ^mut^ / *FLT3*‐ITD ^(−)^/ *TET2* ^(+)^ is an unfavorable prognostic subset in *NPM1* ^mut^ / *FLT3*‐ITD ^(−)^ patients

**FIGURE 2:**
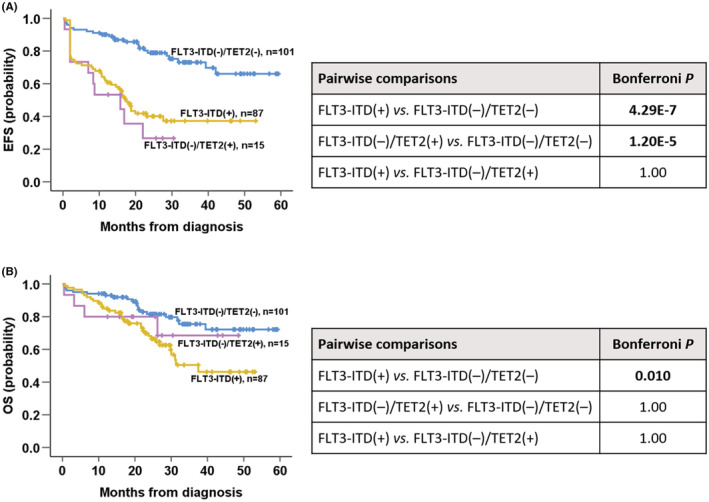
Comparison of EFS (A) and OS (B) across *NPM1* ^mut^/*FLT3*‐ITD^(−)^/*TET2* ^(−)^, *NPM1* ^mut^/*FLT3*‐ITD^(−)^/*TET2* ^(+)^, and *NPM1* ^mut^/*FLT3*‐ITD^(+)^ subgroups. Patients with *NPM1* ^mut^/*FLT3*‐ITD^(−)^/*TET2* ^(−)^ had a superior outcome versus those with *NPM1* ^mut^/*FLT3*‐ITD^(+)^ for all endpoints (Bonferroni: cCR rate *p* = 7.5 E‐05; EFS *p* = 4.29 E‐7; OS *p* = 0.010), for those with *NPM1* ^mut^/*FLT3*‐ITD^(−)^/*TET2* ^(+)^ in terms of the cCR rate (Bonferroni *p* = 0.043) and EFS (Bonferroni *p* = 1.20 E‐5). Patients with *NPM1* ^mut^/*FLT3*‐ITD^(−)^/*TET2* ^(+)^ were comparable to those with *NPM1* ^mut^/*FLT3*‐ITD^(+)^ in terms of all endpoints (Bonferroni *p* = 1.000 for all).

Given the significance of *TET2* mutation, we compared clinical outcomes across the *NPM1* ^mut^/*FLT3*‐ITD^(−)^/*TET2* ^(−)^, *NPM1* ^mut^/*FLT3*‐ITD^(−)^/*TET2* ^(+)^, and *NPM1* ^mut^/*FLT3*‐ITD^(+)^ subgroups. The *NPM1* ^mut^/*FLT3*‐ITD^(−)^/*TET2* ^(−)^ subgroup was superior to *NPM1* ^mut^/*FLT3*‐ITD^(+)^ subgroup in all endpoints (Bonferroni: cCR rate *p* = 7.5 E‐05; EFS *p* = 4.29 E‐7; OS *p* = 0.010; Figure 2A,B), and to the *NPM1* ^mut^/*FLT3*‐ITD^(−)^/*TET2* ^(+)^ subgroup regarding cCR rate (Bonferroni *p* = 0.043) and EFS (Bonferroni *p* = 1.20 E‐5; Figure 2A). There were no differences between *NPM1* ^mut^/*FLT3*‐ITD^(−)^/*TET2* ^(+)^ and *NPM1* ^mut^/*FLT3*‐ITD^(+)^ regarding all endpoints (Bonferroni *p* = 1.000 for all; Figure 2A,B).

### 3.6 Number of mutations in *NPM1* ^mut^ AML independently predicts OS, regardless of *FLT3*‐ITD status

Overall, after adjusting the biased confounders related to induction and transplantation, the independent predictors of OS included number of mutated genes [HR = 1.99 (1.16–3.42), *p* = 0.013], *FLT3*‐ITD [HR = 2.77 (1.59–4.85), *p* < 0.001], and allo‐SCT [HR = 0.42 (0.22–0.81), *p* = 0.010]. Following *FLT3*‐ITD stratification, CD38^(+)^ [HR = 0.23 (0.08–0.65), *p* = 0.005] independently predicted superior OS, and male sex [HR = 3.85 (1.56–9.51), *p* = 0.003] and number of mutated genes ≥4 [HR = 5.54 (1.77–17.33), *p* = 0.003] independently predicted reduced OS in the *NPM1* ^mut^/*FLT3*‐ITD^(−)^ group. In the *NPM1* ^mut^/*FLT3*‐ITD^(+)^ group, ≥5 mutated genes [HR = 2.85 (1.33–6.10), *p* = 0.007] independently predicted reduced OS, and allo‐SCT [HR = 0.45 (0.21–0.94), *p* = 0.033] independently predicted improved OS (Table 4).

**TABLE 4:** Univariate Kaplan–Meier and multivariate Cox analysis of OS in *NPM1* ^mut^ patients stratified by *FLT3*‐ITD.

Group	Factors	Versus	Kaplan–Meier	Cox	
			*p*	HR (95%CI)	*p*
Entire cohort^a^	CD34	(−) vs. (+)	0.065	NS	NS
	No. of mutated genes	<5 vs. ≥5	0.007	***1.99* (*1.16–3.42*)**	***0.013***
	No. of mutated genes	<6 vs. ≥6	0.022	NS	NS
	*FLT3*	(−) vs. (+)	0.009	NS	NS
	*FLT3*‐ITD	(−) vs. (+)	0.005	***2.77* (*1.59–4.85*)**	< ***0.001***
	Induction	SD vs. ID	0.038	NS	NS
	Allo‐SCT	Not vs. Yes	0.075	***0.42* (*0.22–0.81*)**	***0.010***
*NPM1* ^mut^/*FLT3*‐ITD^(−)^	Sex	F vs. M	0.017	***3.85* (*1.56–9.51*)**	***0.003***
	WBC	<45 vs. ≥45	0.039	NS	NS
	CD38	(−) vs. (+)	0.027	***0.23* (*0.08–0.65*)**	***0.005***
	No. of mutated genes	<4 vs. ≥4	0.023	***5.54* (*1.77–17.33*)**	***0.003***
*NPM1* ^mut^/*FLT3*‐ITD^(+)^ ^b^	No. of mutated genes	<5 vs. ≥5	0.001	***2.85* (*1.33–6.10*)**	***0.007***
	No. of mutated genes	<6 vs. ≥6	0.009	NS	NS
	Induction	SD vs. ID	0.019	NS	NS
	Allo‐SCT	Not vs. Yes	0.014	***0.45* (*0.21–0.94*)**	***0.033***

*Note*: Parameters showing statistical significance are highlighted in bold and italic.

Abbreviations: Allo‐SCT, allogeneic stem cell transplantation; CI, confidence interval; F, female; HR, hazard ratio; ID, intermediate‐dose; M, male; NS, not significant; SD, standard‐dose; WBC, white blood cell.

^a^ Adjusted for induction‐associated biased factors.

^b^ Adjusted for transplant‐associated biased factors.

### 3.7 *FLT3*‐ITD combined with induction can further refine EFS stratification of *NPM1* ^mut^ AML

**FIGURE 3:**
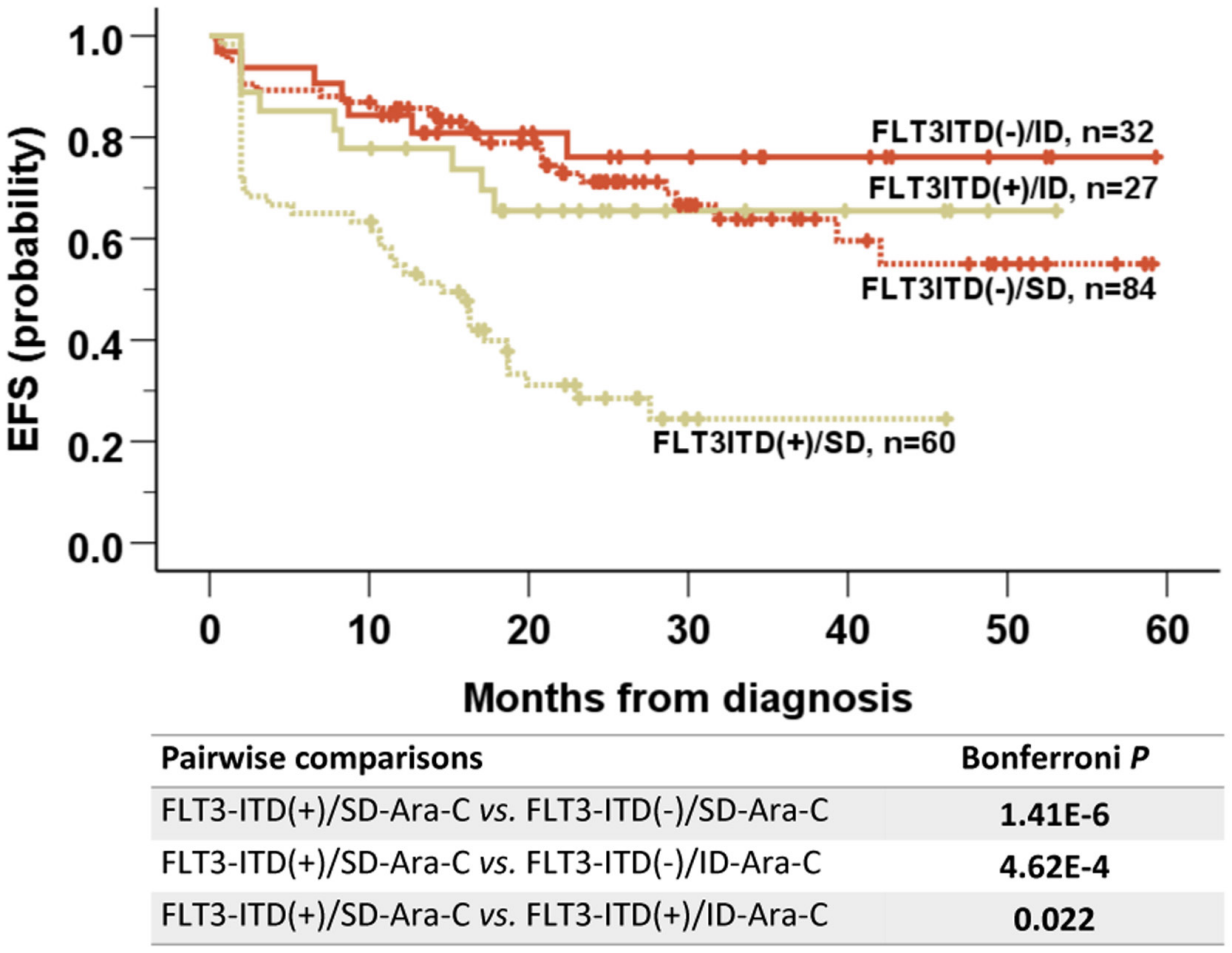
Comparison of EFS between subgroups defined by combination of *FLT3*‐ITD and induction in *NPM1* ^mut^ patients. *FLT3*‐ITD^(+)^/SD‐Ara‐C subgroup had the worst EFS (2‐year EFS rate, 29% ± 6%; 3‐year EFS rate, 24% ± 7%; median EFS, 14.6 months [95% CI 10.3–18.9 months]; Bonferroni *p* < 0.05 for all) when compared with the other three subgroups.

Finally, as *FLT3*‐ITD and induction regimen were independent predictors on EFS in *NPM1* ^mut^ patients, further analysis integrating these two factors further refined risk‐stratification of EFS in *NPM1* ^mut^ patients. The *FLT3*‐ITD^(+)^/SD‐Ara‐C subgroup had the worst EFS (2‐year EFS rate: 29% ± 6%; 3‐year EFS rate: 24% ± 7%; median EFS: 14.6 [95% CI 10.3–18.9] months). The EFS rate was significantly different from the other three subgroups (median EFS not reached, Bonferroni *p* < 0.05 for all; Figure 3). In contrast, the EFS of *FLT3*‐ITD^(+)^/ID‐Ara‐C subgroup was similar to that of the *FLT3*‐ITD^(−)^/SD‐Ara‐C and *FLT3*‐ITD^(−)^/ID‐Ara‐C subgroups (Bonferroni *p* > 0.05 for both; data not shown).

## 4 DISCUSSION

*NPM1* ^mut^ occurs in one‐third of de novo AML and is associated with a good prognosis.^5^, ^6^ Approximately 40% of *NPM1* ^mut^ cases are concurrent with *FLT3*‐ITD, which impairs the favorable effect of *NPM1* ^mut^.^20^, ^21^, ^22^, ^23^, ^24^ Despite the increasing use of *FLT3* inhibitors, the mainstay of the “7 + 3” regimen in treatment of *FLT3*‐ITD^(+)^ AML is still unshakable. In developing countries, FLT3 inhibitors cannot typically be prescribed as conventional drugs due to their lack of accessibility, economic burden, and tolerance by some patients. The correlation between *FLT3*‐ITD^(+)^ with Ara‐C specific resistance^14^, ^15^ prompted the present demonstration that ID‐Ara‐C induction improved the response rate and mitigated induction failure in *NPM1* ^mut^/*FLT3*‐ITD^(+)^ patients. These findings support the notion that Ara‐C uptake deficiency can be partially compensated by increasing the dosage of administered Ara‐C.^14^ Of the four patients with *NPM1* ^mut^/*FLT3*‐ITD^(+)^ who did not attain CR after a first cycle of ID‐Ara‐C induction, re‐induction CR was noted in only one patient, who had previously obtained PR. This suggests that ID‐Ara‐C containing chemotherapy could be a first‐line option for initial induction, in addition to its role as a main component of second‐line rescue protocol. This could allow physicians to identify and ameliorate primary resistance. In this study, ID‐Ara‐C containing induction benefited the cCR rate and EFS only in the *NPM1* ^mut^/*FLT3*‐ITD^(+)^ group, with no benefits in the *NPM1* ^mut^/*FLT3*‐ITD^(−)^ group. Therefore, *NPM1* ^mut^/*FLT3*‐ITD^(+)^ patients can be promisingly induced by initially increasing Ara‐C intensity to improve short‐term efficacy. Moreover, the toxicity of the ID‐ and SD‐Ara‐C regimens were similar and did not increase the risk of induction‐related ED.

Maurillo et al.^25^ combined Ara‐C intensity with MRD in AML patients. The probability of achieving MRD^(−)^ was similar between the HD‐ and SD‐Ara‐C regimens in *NPM1* ^(+)^ or *FLT3*‐ITD^(+)^ patients regardless of whether after induction or during consolidation. When restricting analysis to MRD^(−)^, the authors even revealed the prognostic superiority of the SD‐regimen compared to the HD‐Ara‐C regimen. However, in that study, only patients who achieved morphological CR after induction were selected for survival analysis. This does not exclude the possibility that the outcome analysis included a considerable percentage of patients who reached CR induced with HD‐Ara‐C, but tended to have primary resistance, which cannot be identified by SD‐Ara‐C. Furthermore, more patients who did not respond to SD‐Ara‐C were excluded from the analysis, resulting in an interferential bias. Interestingly, disease‐free survival of HD‐Ara‐C was still better than that of SD‐Ara‐C if cases were confined to continuous MRD^(+)^ during consolidation, suggestive of an inhibition effect of HD‐Ara‐C on resistant leukemic clone to defer the occurrence of relapse and prolong OS.

In addition to passively increasing Ara‐C dosage, the manner of administration of Ara‐C may also affect the chemotherapeutic efficacy.^25^ In this study, the first 4 days of the induction schedule of the ID‐Ara‐C regimen was the same as that of the SD‐Ara‐C regimen, with ID‐Ara‐C supplemented in days 5–7. Adding Ara‐C dosage at the later stage of induction schedule has several advantages. First, for patients with hyperleukocytosis, later administration of ID‐Ara‐C can reduce risk of tumor lysis syndrome. Second, there is the opportunity to wait for a turnaround of the *FLT3*‐ITD results before deciding whether to adjust the Ara‐C dose. Although the present findings indicate the improvement of ID‐Ara‐C in *NPM1* ^mut^/*FLT3*‐ITD^(+)^ patients, we assume that this effect may otherwise be extended to all *FLT3*‐ITD^(+)^ patients regardless of *NPM1* ^mut^ status. This assumption needs to be confirmed.

*FLT3*‐ITD did not change the degree of DNA damage after daunorubicin chemotherapy, but was linked with an increased level of p53. *FLT3*‐ITD^(+)^ leukemic cells were desensitized in the presence of p53, while chemosensitivity was restored after knockout of *TP53*, indicating a dependence on p53 for the resistance of *FLT3*‐ITD^(+)^ leukemia cells.^26^ Therefore, the combination of SD‐Ara‐C with daunorubicin may be suboptimal for induction.^11^ An alternative of anthracyclines, such as idarubicin (IDA) substituting for DNR, might be considered to potentially improve the efficacy. If applicable, *FLT3* inhibitor can be administrated as soon as possible to sensitize cytotoxic chemotherapy and mitigate resistance.^26^, ^27^ However, in our study, owing to the non‐homogenous usage of *FLT3* inhibitor, varying agent selection, and initiation timing and duration, it was not a covariate in multivariable outcome analysis in the *NPM1* ^mut^/*FLT3*‐ITD^(+)^ group.

Among the comutations of *NPM1* ^mut^ AML, *TET2* ^(+)^ was identified as an independent factor predicting lower cCR rate and inferior EFS in the *NPM1* ^mut^/*FLT3*‐ITD^(−)^ group, consistent with other results.^28^, ^29^ Moreover, integration of *TET2* and *FLT3*‐ITD could better discriminate patients with *NPM1* ^mut^ AML into three prognostic subsets, with *NPM1* ^mut^/*FLT3*‐ITD^(−)^/*TET2* ^(+)^ comparable to *NPM1* ^mut^/*FLT3*‐ITD^(+)^ group regarding all outcome endpoints. Our findings differ from the study of Chou et al.,^30^ who reported no significant impact of *TET2* mutation on OS among AML patients with favorable molecular genotypes (*NPM1* ^mut^/*FLT3*‐ITD^(−)^ or *CEBPA* ^double^). However, the survival curve indicated a trend of OS difference between the *TET2* wild and mutant subgroups, although not significant (*p* = 0.198). The lack of significance may be due to the limitations of the number of cases and ethnic disparity. Moreover, the authors combined *NPM1* ^(+)^/*FLT3*‐ITD^(−)^ and CEBPA^double^, and did not analyze the specific *NPM1* ^(+)^/*FLT3*‐ITD^(−)^ individually.

According to guidelines,^7^, ^8^ AML having the *NPM1* ^mut^ without *FLT3*‐ITD or with *FLT3*‐ITD^low^ genotype were combined and allocated into a favorable‐risk. However, the AMLSG large cohort study^20^ risk‐stratified patients as per the 2017‐European LeukemiaNet criteria, demonstrating that OS of *NPM1* ^mut^/*FLT3*‐ITD^low^ group <60 years of age was worse than that of other favorable‐risk groups (*p* = 8.7 × 10^−8^) including *NPM1* ^mut^/*FLT3* ^wt^ cases. The OS rate did not significantly differ from that of intermediate‐risk group (*p* = 0.76), which included *NPM1* ^mut^/*FLT3*‐ITD^high^ AR cases. The survival rates in patients ≥60 years of age were also similar across *NPM1* ^mut^/*FLT3* ^wt^, *NPM1* ^mut^/*FLT3*‐ITD^low^, and *NPM1* ^mut^/*FLT3*‐ITD^high^. Additionally, in the small cohort of The Cancer Genome Atlas data, *NPM1* ^mut^/*FLT3*‐ITD^low^ had similar poor OS with *NPM1* ^mut^/*FLT3* ^wt^ patients because of higher median age in both groups. The authors concluded that patients with *NPM1* ^mut^/*FLT3*‐ITD^low^, as well as those with *NPM1* ^mut^/*FLT3* ^wt^ ≥ 60 years of age, could not be classified into a favorable‐risk. Other studies also demonstrated no significant prognostic difference between patients with *NPM1* ^mut^/*FLT3*‐ITD^low^ and *NPM1* ^mut^/*FLT3*‐ITD^high^.^21^, ^22^, ^23^, ^24^ Thus, it appeared reasonable to re‐allocate *NPM1* ^mut^/*FLT3*‐ITD^low^ to an intermediate‐risk. This is embodied in the very latest published recommendations, 2022 European LeukemiaNet risk classification by genetics at initial diagnosis in AML, which categorizes *NPM1* ^mut^/*FLT3*‐ITD as intermediate‐risk irrespective of *FLT3*‐ITD AR.^31^

CD34^(+)^ was associated with primary resistance and poorer EFS in *NPM1* ^mut^/*FLT3*‐ITD^(+)^ group, similar to other findings.^32^, ^33^ The combination of varying features of antigen expression is more informative in predicting survival, where CD34^(+)^/CD38^(−)^/CD123^(+)^ representing the leukemia stem cell phenotype has prognostic relevance.^34^ Chen et al.^35^ characterized the antigen expression among 94 *NPM1* ^mut^ patients by cluster analysis and divided them into two categories according to CD34, CD7, and HLA‐DR. The results revealed a significantly unfavorable prognosis for type‐II features characterized by CD34^(+)^/HLA‐DR^(+)^/CD7^(+)^ compared to type‐I features characterized by CD34^(−)^/CD7^(−)^. Mason et al.^36^ analyzed myeloid blast populations excluding monocytic differentiation in *NPM1* ^mut^ patients. The acute promyelocytic leukemia‐like phenotype CD34^(−)^/HLA‐DR^(−)^/MPO^(str+)^ was present in nearly half the patients (48%) and beneficially influenced RFS and OS. Therefore, the leukemic immunophenotypes should not be limited to AML diagnosis, but rather additionally play roles in prognostication and MRD monitoring, to guide treatment.

In the present study, AR was detected in only some *NPM1* ^mut^/*FLT3*‐ITD^(+)^ patients, so we did not include *FLT3*‐ITD AR in the analysis. A recent study concerning the relationships between CD34 with *NPM1* ^mut^ or *FLT3*‐ITD described the distribution of *FLT3*‐ITD^high^ almost exclusively in the CD34^(+)^ group and rarely in the CD34^(−)^ group, the latter entirely presenting *FLT3*‐ITD^low^ features.^37^ Thus, CD34 negativity may likely imply an absence of *FLT3*‐ITD or low AR, which partly explains the better prognosis of CD34^(−)^ patients than those with CD34^(+)^. Our findings indicate that ID‐Ara‐C as a first‐line induction in *NPM1* ^mut^/*FLT3*‐ITD^(+)^ patients improved the remission rate, especially for CD34^(+)^ patients at the initial diagnosis. CD34 in *NPM1* ^mut^/*FLT3*‐ITD^(−)^ was not prognostically significant, partly due to a lower CD34^(+)^ percentage and the accordingly reduced occurrence of endpoints, with no statistical significance. EFS stratification of *NPM1* ^mut^ AML could be further refined by the combination of *FLT3*‐ITD with an induction scheme. *FLT3*‐ITD^(+)^/SD‐Ara‐C displayed the worst EFS. This finding indicates that *NPM1* ^mut^/*FLT3*‐ITD^(+)^ patients can be initially induced by ID‐Ara‐C, especially CD34^(+)^ patients, who tended to be enriched for *FLT3*‐ITD^high^ characteristics.

Consistent with other results,^38^ the number of mutated genes at initial presentation in our *NPM1* ^mut^ cohort independently predicted OS, regardless of *FLT3*‐ITD and induction intensity, highlighting an interplay between coexisting genetic lesions. A Japanese report showed that even patients with *NPM1* ^mut^/*FLT3*‐ITD^(−)^ presented an inferior outcome when a high number of mutations coexist.^39^ This finding highlights the broad coverage and high throughput value of NGS for determining genetic alterations.

In our cohort, some traditional indicators, such as increasing age and high WBC count, had independent impacts on EFS in *NPM1* ^mut^/*FLT3*‐ITD^(−)^ patients, similar to other results.^20^, ^23^, ^40^ These “old” parameters may be a comprehensive embodiment of the external effects of varying biological mechanisms intrinsically harbored in leukemia cells. For example, WBC reflects leukemic burden and cell proliferation and viability, and age reflects a distribution tendency toward clonal hematopoiesis‐related mutations, as well as performance status and organic comorbidity, which decrease chemotherapeutic tolerance.

There were several limitations in our study. First, this is a retrospectively nonrandomized study design. Its inherent selection bias leads to potential confounders (e.g., divergent salvage protocols and FLT3 inhibitor usage, supportive care heterogeneity), although the analyzed cases received intensive therapy with a curative intent. A second limitation is missing data on immunophenotypic markers, which might compromise the statistical power of other covariates in a multivariate model. Although the transplant‐associated biases were adjusted in the survival analysis, discrepant transplant indications, and timing and donor‐recipient HLA relationships were present. Also, loss to follow‐up for some cases might affect study results. Future prospective studies are necessary to confirm the present findings.

To summarize, patients with *NPM1* ^mut^ AML have differential predictors according to their *FLT3*‐ITD status. *TET2* mutation, age, and WBC count are independently associated with outcome in AML with *NPM1* ^mut^/*FLT3*‐ITD^(−)^. Positive CD34 expression is adversely associated with outcome in AML with *NPM1* ^mut^/*FLT3*‐ITD^(+)^ where initial ID‐Ara‐C induction and allo‐SCT consolidation can benefit efficacy. The number of mutated genes predicts OS, regardless of *FLT3*‐ITD status. We identified high‐risk subsets among *NPM1* ^mut^ AML patients, allowing individually‐tailored management strategies in this AML subtype.

## AUTHOR CONTRIBUTIONS

**Biao Wang:** Data curation (equal); formal analysis (equal); software (equal); writing – original draft (equal); writing – review and editing (equal). **Xiaoying Hua:** Resources (equal); validation (equal); visualization (equal); writing – review and editing (equal). **Jihong Zhang:** Data curation (equal); methodology (equal); resources (equal); writing – review and editing (equal). **Weiying Gu:** Conceptualization (equal); funding acquisition (equal); supervision (lead); writing – review and editing (equal). **Haiqian Li:** Supervision (supporting); validation (equal); writing – review and editing (equal).

## CONFLICT OF INTEREST STATEMENT

The authors declare that they have no conflict of interests.

## Supporting information


Table S1

Table S2

## Data Availability

The datasets used and/or analyzed during the current study are available from the corresponding author upon reasonable request.
