# Supplementary material for: Prognostication refinement in NPM1‐mutated acute myeloid leukemia stratified by FLT3‐ITD status with different induction doses of cytarabine
Source: Cancer Med. 2023 Feb 21;12(8):9420–33. doi: 10.1002/cam4.5704 (PMC10166952; doi:10.1002/cam4.5704)
Supplement: Supplementary file 1 — Table S1 [file CAM4-12-9420-s002.docx]

# Table S1. Bias test of induction-related variables for cCR rate in entire *NPM1*^mut^ cohort and in groups divided by *FLT3*-ITD

| **Group** | **Factors** | **Entire** | **SD group** | **ID group** | ***P*#** |
| --- | --- | --- | --- | --- | --- |
| Whole | No. of patients | N=196 | N=138 | N=58 | NA |
|  | Sex (M:F), N | 86:11 | 66:72 | 20:38 | 0.086 |
|  | Median age (range), y | 48 (15-69) | 51 (15-66) | 43 (19-69) | <0.001 |
|  | *TET2*, n (%) | 28 (14.3) | 24 (17.4) | 4 (6.9) | 0.055 |
|  | *KMT2D*, n (%) | 21 (10.7) | 10 (7.2) | 11 (19.0) | 0.015 |
| *FLT3*-ITD(–) | No. of patients | N=112 | N=81 | N=31 | NA |
|  | Median age (range), y | 49 (16-69) | 51 (16-66) | 44 (24-69) | 0.015 |
| *FLT3*-ITD(+) | No. of patients | N=86 | N=59 | N=27 | NA |
|  | Sex (M:F), N | 44:42 | 37:22 | 7:20 | 0.002 |
|  | Median age (range), y | 46 (15-65) | 50 (15-65) | 42 (19-54) | 0.003 |
|  | Median Hb (range), g/L | 87.5 (49-154) | 89 (51-154) | 79 (49-128) | 0.031 |
|  | CD34, n/N (%) | 40/85 (47.1) | 23/58 (39.7) | 17/27 (63.0) | 0.045 |
|  | *PTPN11*, n (%) | 5 (5.8) | 1 (1.7) | 4 (14.8) | 0.055C |
|  | *TET2*, n (%) | 14 (16.3) | 13 (22.0) | 1 (3.7) | 0.068C |
|  | *KMT2D*, n (%) | 9 (10.5) | 3 (5.1) | 6 (22.2) | 0.042C |

**Abbreviations:** SD, standard-dose; ID, intermediate-dose; *P*#, *P*-values between the SD- and ID-Ara-C groups; NA, not applicable; C, continuity correction
