# Supplementary material for: Prognostication refinement in NPM1‐mutated acute myeloid leukemia stratified by FLT3‐ITD status with different induction doses of cytarabine
Source: Cancer Med. 2023 Feb 21;12(8):9420–33. doi: 10.1002/cam4.5704 (PMC10166952; doi:10.1002/cam4.5704)
Supplement: Supplementary file 2 — Table S2 [file CAM4-12-9420-s001.docx]

# Table S2. Bias test of induction-related variables for EFS and OS in entire *NPM1*^mut^ cohort and in groups divided by *FLT3*-ITD

| **Group** | **Factors** | **Entire** | **Induction** | | ***P*#** |
| --- | --- | --- | --- | --- | --- |
|  |  |  | **SD group** | **ID group** |  |
| Whole | No. of patients | N=203 | N=144 | N=59 | NA |
|  | Sex (M:F), N | 89:114 | 69:75 | 20:39 | 0.068 |
|  | Median age (range), y | 48 (15-69) | 51 (15-66) | 43 (19-69) | <0.001 |
|  | *KMT2D*, n (%) | 22 (10.8) | 11 (7.6) | 11 (18.6) | 0.022 |
| *FLT3*-ITD(–) | No. of patients | N=116 | N=84 | N=32 | NA |
|  | Median age (range), y | 49 (16-69) | 51 (16-66) | 44.5 (24-69) | 0.011 |
| *FLT3*-ITD(+) | No. of patients | N=87 | N=60 | N=27 | NA |
|  | Sex (M:F), N | 44:43 | 7:20 | 37:23 | 0.002 |
|  | Median age (range), y | 46 (15-65) | 49.5 (15-65) | 42 (19-54) | 0.004 |
|  | Median Hb (range), g/L | 87 (49-154) | 88.5 (51-154) | 79 (49-128) | 0.036 |
|  | CD34, n/N (%) | 40/86 (46.5) | 23/59 (39.0) | 17/27 (63.0) | 0.039 |
|  | *PTPN11*, n (%) | 5 (5.7) | 1 (1.7) | 4 (3.7) | 0.052C |
|  | *TET2*, n (%) | 14 (16.1) | 13 (21.7) | 1 (3.7) | 0.073C |
|  | *KMT2D*, n (%) | 9 (10.3) | 3 (5.0) | 6 (22.2) | 0.039C |

**Abbreviations:** SD, standard-dose; ID, intermediate-dose; *P*#, *P*-values between the SD- and ID-Ara-C groups; NA, not applicable; C, continuity correction
